# Digital marketing in attracting new patients: cross-sectional study in a vascular surgery office

**DOI:** 10.1590/1677-5449.202500422

**Published:** 2025-12-15

**Authors:** Alex Lazzari Dornelles, Fabricio Rodrigues Santiago, Graciela Aparecida Pelegrini, Camila Biedler Giordani, Thais Muraro Simionato, Silvana Klaki Ternopilski, Mariana Mello

**Affiliations:** 1 Angioclínica, Chapecó, SC, Brasil.; 2 Clínica Longevittá – Angioclínica, Maravilha, SC, Brasil.; 3 Universidade Federal de Goiás – UFG, Goiânia, GO, Brasil.; 4 Instituto Federal de Santa Catarina – IFSC, Chapecó, SC, Brasil.; 5 Universidade de Passo Fundo – UPF, Passo Fundo, RS, Brasil.; 6 Universidade Federal do Rio Grande do Sul – UFRGS, Porto Alegre, RS, Brasil.

**Keywords:** social media, health, health services marketing, costs and cost analysis, medical informatics

## Abstract

**Background:**

Over the years and the evolution of technology, social networks have become widely accessible and play an important role in interpersonal relationships. Currently, the use of these platforms to disseminate health information ranging from diagnosis to treatments and medical procedures is increasing. In this sense, this study aimed to verify the importance of using social networks in attracting new patients to the office.

**Objectives:**

This study aimed to verify the impact of using social networks on attracting new patients in a medical office of angiology and vascular surgery.

**Methods:**

The study was carried out in a private vascular surgery office, with a sample of 604 patients. Data was collected from January 2022 to December 2023 through an electronic registration, filled out by the secretary at the office.

**Results:**

From the total of 604 patients, 77% were female and 23% male. Of the total number of patients, 41% found out about the office through advertisements made on social media. This generated a positive impact of 38% on the practice’s revenue in the period analyzed.

**Conclusions:**

Analysis of the results indicated that the office's presence on social media contributed positively to attracting new patients, as well as increasing revenue. Digital marketing is a trend that will only grow with the spread of the internet and including social networks in the dissemination of health information generates benefits for both parties: doctor and patient.

## INTRODUCTION

Marketing is an important tool in many areas that can be used to promote goods, services, events, experiences, people, places, properties, organizations, information, and ideas.^1^ As computers and information technology became ubiquitous, marketing began to turn its attention to the internet, giving rise to digital marketing (DM).^2^

Digital marketing is selling products and services via digital formats^3^ and, in the area of health, it has become a factor of differentiation in service provision, enabling professionals, including physicians, to promote their services to people all over the world, and not just to those in the vicinity of their offices.^4,5^ In turn, this allows promotion of their work to keep pace with current trends in the digital scenario.

Now that the internet, social media, mobile applications, and other technologies for digital communication are integrated into daily life, billions of people all over the world use these tools daily,^6^ meaning that an online presence is important for medical clinics to achieve visibility and attract new patients. In line with this, there has been a notable increase in use of digital communication tools and social media by different health professionals, especially physicians.^7^

Since the emergence of social media, a growing percentage of patients have been using these technologies for health-related reasons.^8,9^ In this scenario, publicity on social media constitutes a field in expansion in many different areas of medicine.^10-12^

Nowadays, there are many different social media platforms, with different styles of utilization.^13^ These include, Instagram®, Facebook®, X® (formerly Twitter®), YouTube®, blogs, and on-line health communities, which are popular among patients and are used to search for and share information.^14-16^ Moreover, use of these platforms can facilitate patient involvement, outreach to the community in general, and peer-to-peer education and learning.^12^

Social media are considered influential factors in physician-patient relationship and use of DM optimizes patient involvement, impacting their choices and often leading to purchases or subscriptions to products or services.^17,18^

The interactions between physician and patient that are enabled by social media facilitate exchange of information and collaborative content creation. In other words, the physician and the patient work together to ensure that the information that is posted on social media is more precise.^14,15,19^

In view of the above, the overall objective of this article was to verify the impact of use of social media on enrollment of new patients at an angiology and vascular surgery clinic. Specific objectives were to investigate how patients found out about the clinic and calculate the proportion of the clinic’s revenue generated by patients who sought the clinic because of social media. The relevance of the study lies in the fact that the area investigated is highly specialized, which is why patients very often need detailed and trustworthy information when choosing a professional or clinic.

## METHOD

**Figure 1:**
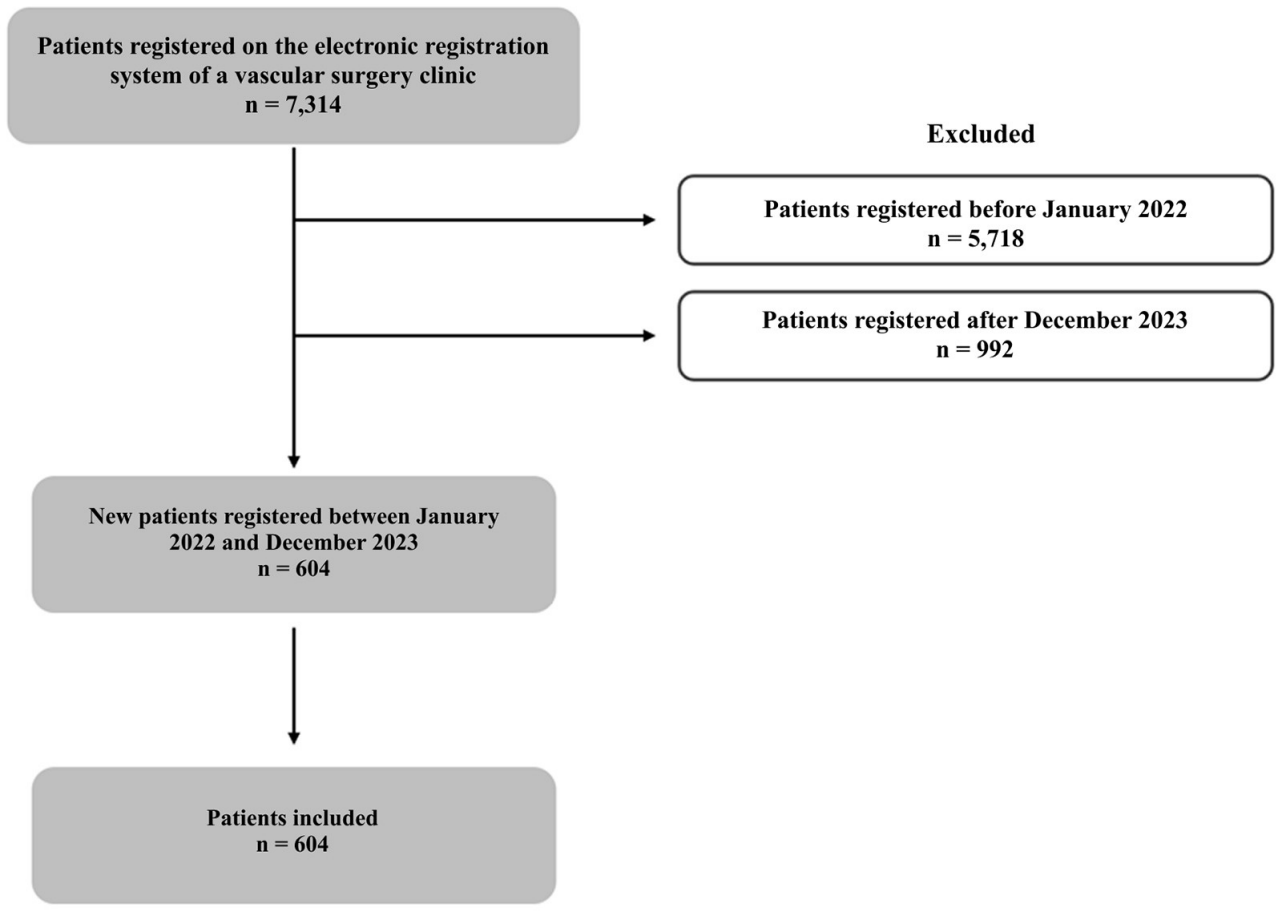
Flow diagram illustrating the study design.

This is a retrospective cross-sectional study of data acquired prospectively. Data were collected by quantitative cross-sectional survey. Data were extracted from patients’ electronic registration forms. Overall, data were collected on 604 patients from January 2022 to December 2023. The inclusion criterion was all new patients enrolled on the clinic’s electronic registration system from January 2022 to December 2023. Exclusion criteria were patients who were registered before 2022 (n = 5,718) or after December 2023 (n = 992). Figure 1 shows the study flow diagram.

The ideal sample size was calculated based on a 95% confidence level, 5% margin of error, and an estimated proportion of 41%, resulting in a minimum sample size of 372 patients. Since the study analyzed a sample of 604 patients, the sample size was considered adequate.^20^

The clinic’s DM strategy uses Instagram®, Youtube®, and the clinic’s own website. The clinic places paid content on Instagram®, where it had 232 thousand followers in October 2024. The clinic’s Youtube® channel has more than 390 thousand subscribers.

This clinic was chosen for the study because it met the following pre-established criteria: having a social media presence; publishing actively; reaching at least 200 thousand people via these media; and having been in business in the city for at least 5 years. Microsoft Excel® and the Statistical Package for the Social Sciences were used to tabulate and analyze data.

This study was not submitted for Research Ethics Committee (REC) appraisal because, according to Resolution nº 510, “research using information stored in databases in aggregated form that does not enable identification of individuals” does not have to be registered on the National Research Ethics Council REC system (BRAZIL, 2016, Art. 1, Clause 1, V), which is applicable to this study, since the information from the database was accessed in an aggregated form that did not enable identification of patients. The study follows the Strengthening the Reporting of Observational Studies in Epidemiology (STROBE) guidelines.^21^

## RESULTS

**Figure 2:**
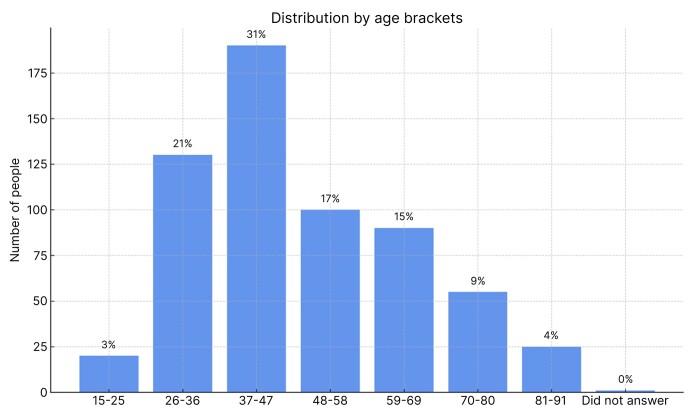
Age of participants.

The clinic analyzed in this study opened for business in 2014 and, over the last 3 years, has been seeing 18 patients per day. The clinic is open from Wednesday to Friday, for consultations, follow-up consultations, and procedures. During the study period, the clinic was only serving private patients and clients from a single health insurer. Data were analyzed on all 604 new patients who were registered during the study period. Figure 2 illustrates these patients’ ages.

The mean age of the whole sample was approximately 49 years. By sex, the mean age of the women in the sample was 48 years, while the mean age of the men was 51 years. The male patients’ ages ranged from 15 to 85 years and the women were aged from 19 to 89 years.

**Figure 3:**
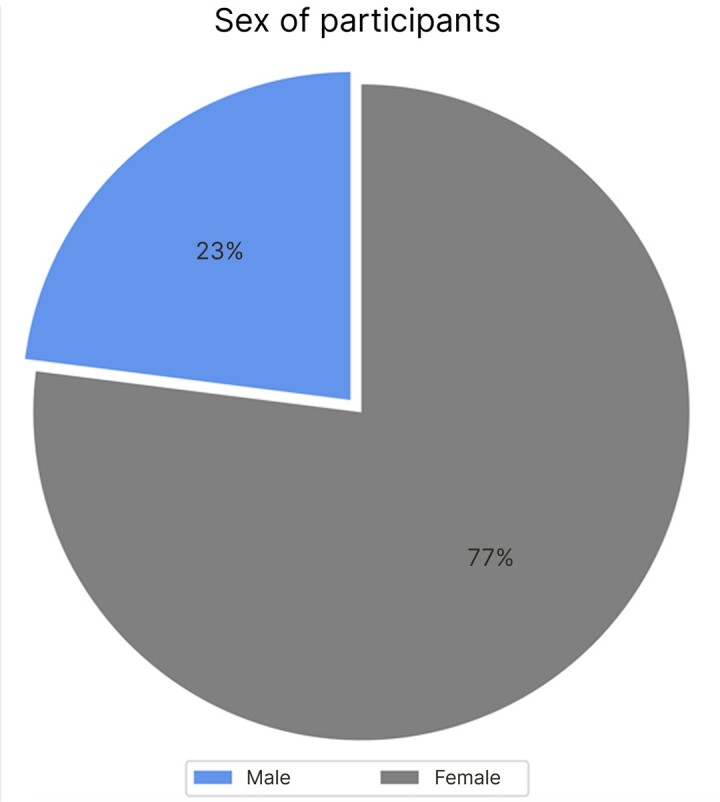
Sex of participants (%).

Figure 3 illustrates the proportion of men and women in the study sample. A total of 466 women and 138 men were registered. These patients stated how they had come to know about the clinic, as shown in Table 1.

**Table 1:** Origins of new patient referrals.

**Origin**	**2022**	**2023**	**Total**
Social media	129	118	247
Friends/relatives	116	112	228
Other physicians	44	53	97
Health insurer	13	7	20
Others	10	2	12
**Total**	312	292	604

Out of 604 new patients, 247 (41%) had found out about the clinic via social media, 228 (38%) had been recommended the clinic by friends or relatives, 97 (16%) had been referred by other physicians, and just 20 (3%) had come via their health insurer. A further 12 patients had learnt about the clinic in other ways that were not specified.

Table 2 lists data on the patients who had been attracted to the clinic by social media. It was observed that a majority of the patients who came to the clinic from social media were aged up to 58 years. Patients aged 59 to 80 years were less likely to be reached by information posted on the clinic’s social media.

**Table 2:** Details of new patients and the proportion attracted by social media.

**Age group**	**Total in sample**	**Social media**	**% recruited via social media in each age group**
15 to 25 years	18	9	50.00
26 to 36 years	129	61	47.29
37 to 47 years	188	80	42.55
48 to 58 years	100	43	43.00
59 to 69 years	91	29	31.87
70 to 80 years	52	16	30.77
81 to 91 years	25	9	36.00
Did not answer	1	0	0.00
Total	604	247	

Out of the entire sample, 156 patients were aged 60 or over. Eight (1.32%) of these patients had been referred to the clinic by their health insurer, 55 (9.10%) had been recommended to seek the clinic by friends or family, 50 (8.27%) came from social media, 38 (6.29%) were referred by other physicians, and five (0.82%) were unable to say how they had found out about the clinic.

**Figure 4:**
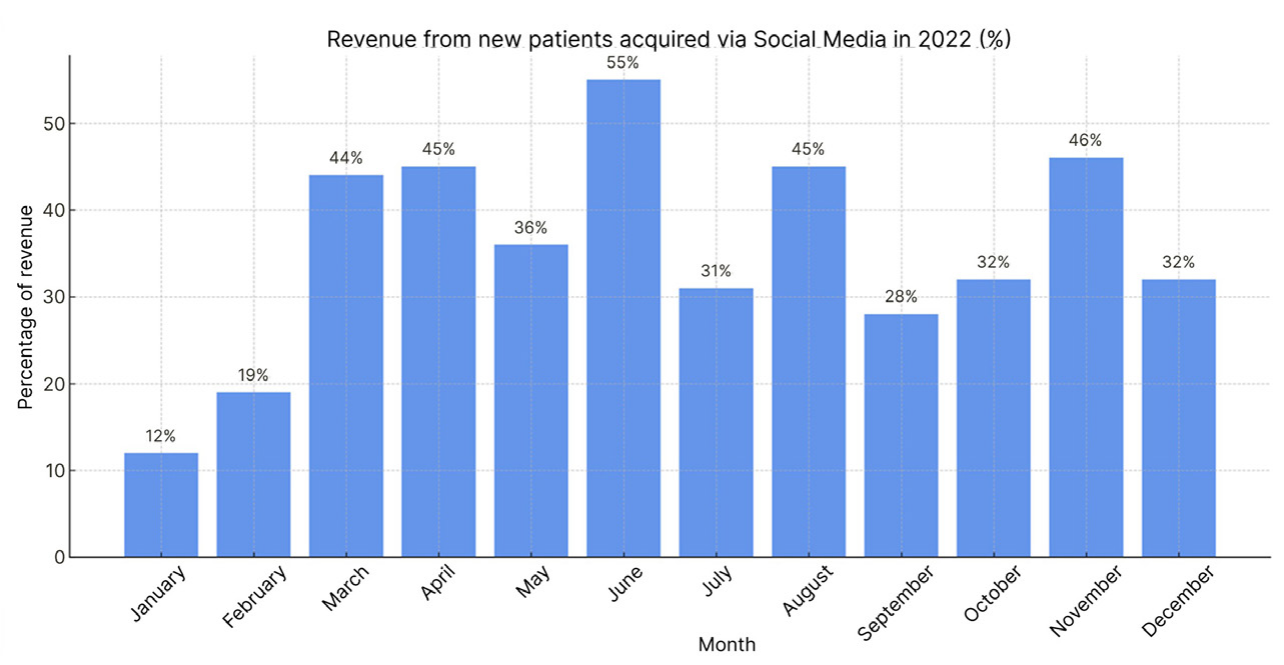
Revenue from patients attracted via social media in 2022 (%).

An analysis was conducted to calculate the proportion of the clinic’s monthly revenue from January 2022 to December 2023 that had come from patients who sought the clinic because of social media. Figure 4 presents the data corresponding to the year 2022.

In 2022, the clinic registered 312 new patients, 129 (41.34%) of whom had come from social media. That year, the mean proportion of the clinic’s revenue due to patients from social media was 35%.

**Figure 5:**
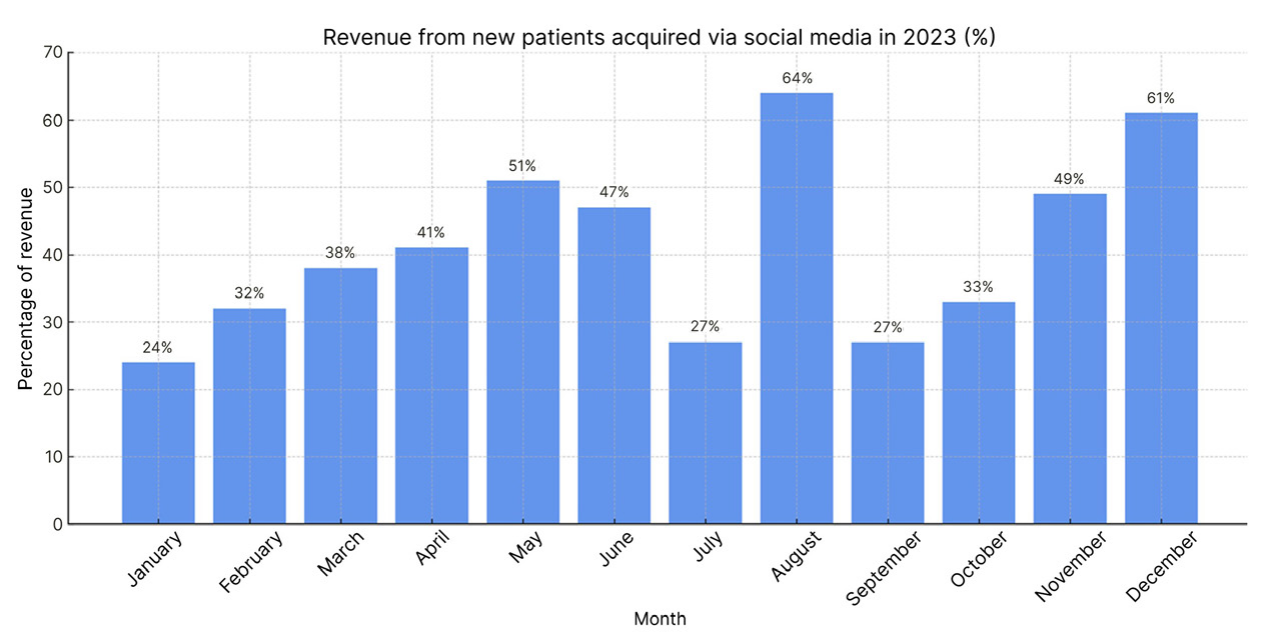
Revenue from patients attracted via social media in 2023 (%).

Figure 5 illustrates the data from 2023, when the clinic registered 292 new patients, 118 of whom came from social media and who accounted for 41% of revenue, on average.

## DISCUSSION

**Figure 6:**
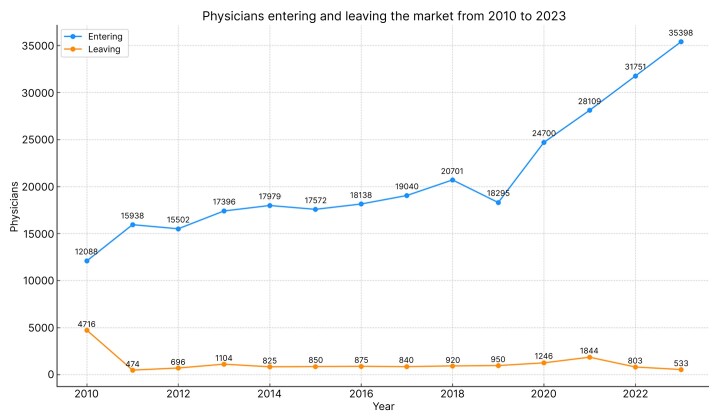
Trend of physicians entering and leaving the market.

The health care industry in Brazil grew significantly over the last decade and the number of physicians entering the market significantly outnumbered the number of professionals who stopped practicing.^22,23^ The total population of physicians in Brazil increased from 304,406 in 2010 to 575,930 in 2024 and it is predicted that this number will pass the 1 million mark in 2035, according to data from the Brazilian medical demographics report.^23^Figure 6 illustrates the numbers of physicians entering and leaving the market each year from 2010 to 2023.

This scenario of growing professional competition in the area,^24^ in conjunction with greater use of the internet and social media by the Brazilian population,^25,26^ creates an environment in which use of DM in the health care industry is ideal.

A recent national survey^25^ found that 93.4% of Brazilians use the internet every day to make voice or video calls (94.4%), send or receive messages (92.0%), watch videos (88.3%), and use social media (83.6%), among other uses. In January of 2024, Brazil’s 187.9 million internet users were spread across many different platforms. Table 3 lists the numbers of users of each of the three largest social media platforms and the reach of advertising on these platforms.

**Table 3:** Number of users and advertising reach of the main social media platforms used in Brazil in 2024.

**Social media platform**	**Users**	**Reach**	**Women**	**Men**
	**(millions)**	**(%)**	**(%)**	**(%)**
YouTube	144.0	66.3	51.4	48.6
Instagram	134.6	62.0	58.4	41.6
Facebook	111,3	51.3	53.8	46.2

Source: Digital Data Report.^26^

According to a published report, Youtube®, Instagram®, and Facebook® are the social media most used in Brazil. Women account for more than half of these networks’ users, with significant advertising reach. The reach of advertising is calculated and shown as a percentage of the total population of Brazil.^25^

For many people, using social media has become a significant part of daily life and the primary source of information.^24-26^ This highlights the importance of using the internet for marketing communication, especially to reach consumers who use social media as a means of obtaining information, communicating, expressing themselves, and constructing relationships.^27,28^

People are increasingly using the internet to seek information, schedule consultations, and find out more about physicians and their specialties,^24,29^ and social media is the preferred platform for accessing information.^30^ These factors highlight the importance for health professionals, especially physicians, of having a presence on social media. Moreover, the growing use of media in people’s daily lives^31^ and their influence on consumer behavior through DM^3^ culminate in consumption of products and services.^18^

Over recent years, health professionals have been increasingly turning to social media to communicate with their patients and promote health.^7,32^ Use of DM via social media enables professionals to demonstrate their competence to potential patients, expand their area of activity, improve the image of the health care industry in general, reach a wider audience, and offer personalized care.^5,33^

While physicians are increasingly using social media, many are still hesitant to adopt the practice.^32^ Recent studies^9,12-14,30,32,34^ conducted in different areas of medicine have investigated use of social media by physicians, highlighting the positive aspects. Among the factors identified are facilitation of patient-physician communication, the capacity to expand physicians’ publicity, and acceleration of health promotion.

Analyzing the data from the study sample, it was observed that the most common ways that patients found out about the clinic were via social media and from friends or relatives (word of mouth). On this basis, it can be stated that social media offers patients a platform from which they can start searching for a professional.^30^

A presence on social media enables physicians to directly address an enormous group of patients,^10^ but, since not all platforms are the same and usage varies from generation to generation, it is important to understand the profile of the patients. Effective marketing via social media should consider content delivery and the correct choice of platform, which are variables that depend on the specific age group of the public the marketing is intended to reach.^12^

The study sample included 466 women, 195 (41.85%) of whom found the clinic on social media. In turn, 52 (37.68%) of the 138 men found out about the clinic via social media. The fact that female clients chose the clinic because of social media more frequently than males may be related to a survey conducted recently,^26^ which found that women comprised more than half of users, outnumbering men. It is possible that women may be more receptive than men or even actively seek to interact with their physicians on social media.^34^ In the case of specialties that primarily serve female patients, this is a very relevant observation.

With regard to age groups, it was observed that patients aged from 59 to 80 years were less likely to be reached directly by information posted on the clinic’s social media. These older patients may be influenced by younger people who have accessed information or found the clinic via social media, since younger generations have grown up in an environment in which digital media have become widespread.^35^

According to data on use of internet in Brazil published by the Brazilian Institute of Geography and Statistics (IBGE - Instituto Brasileiro de Geografia e Estatística),^25^ during 2022, 62.1% of seniors (60 or older) were using the internet. This reveals an opportunity in the form of an audience that the clinic could reach via DM.

The data collected could be used to personalize content for the desired age group being targeted. In this respect, other authors^12^ have found that younger generations are more likely to use Instagram®, Snapchat®, and TikTok®, while older generations may be more likely to use Facebook® and YouTube®^12^ to seek information about health.

Analysis of the data also revealed that use of DM had a significant impact on the clinic’s revenue, via its presence on Instagram®, Youtube®, and its own website. During the period analyzed, the clinic only served private clients and patients from one health insurer. Of these, those attracted by social media accounted for 35% of the clinic’s revenue in 2022 and 41% in 2023. Although the study analyzed a short period of time, it was observed that there was a constant increase in these figures.

The contribution made by social media may actually be even greater if one considers that information posted by the physician on Instagram®, Youtube®, and the clinic’s own website may mean that patients arrive at the clinic with greater knowledge and more predisposed to purchase products or services. This would be because of the trust they have built up in the physician in response to all of the information he has posted on social media.

Corroborating this, other authors^17^ have stated that, with regard to health-related content, people express concern about the legitimacy of information its sources and, as a result, tend to place more trust in information provided by physicians or health professionals, rather than random misleading information.

The numbers presented confirm the financial benefits of using social media, which also increases physician’s publicity.^10,12-14,30^ Beyond the direct financial impact of the new patients attracted by social media, it is also necessary to consider the savings involved if conventional publicity campaigns are compared with campaigns via social media.^10^

With reference to the study sample, even though the sample employed was robust, the ideal sample size was calculated regardless, based on a 95% confidence level, 5% margin of error, and estimated proportion of 41%.^20^ The result indicated that the minimum number of participants needed to guarantee the representativeness of the data would be 372 patients. Since the study sample comprised 604 participants, the number of patients exceeded the minimum sample size, increasing the reliability of the findings and reinforcing the validity of the study conclusions. Therefore, the results presented are a faithful reflection of the positive impact that social media have on acquisition of new patients by angiology and vascular surgery clinics.

Finally, while this study has demonstrated the positive impact of DM on patient acquisition and on the clinic’s revenue, it is essential to take into consideration the ethical issues related to medical advertising.^36,37^ According to the Medical Ethics Code, publicity must be employed responsibly, avoiding sensationalism, self-promotion, and creation of unrealistic expectations.^37^ Publicity via social media should prioritize information based on scientific evidence, respecting patients’ privacy and the prevailing regulations.^36,37^ When conducted ethically and with transparency, DM is capable not only of bringing physicians and patients together, but also of promoting health education and building trust in the relationship between client and professional.

## CONCLUSIONS

As competition in the health care sector has increased, use of social media for DM has become an essential strategy for clinics to acquire new patients and build visibility. This study found that 41% of new patients at the clinic analyzed had been attracted by these platforms, demonstrating the significant impact of DM. Additionally, social media also contributed to a mean increase of 38% in the clinic’s revenue.

While these results are promising, it is important to consider the study’s limitations, since it was conducted at a private vascular surgery clinic, where patients’ social media usage habits may not be generalizable. However, DM has a promising future in health care, with opportunities for innovation and growth that can be financially beneficial for physicians and increase recognition of their brands. Finally, in the health care sector it is indispensable to conduct DM ethically and responsibly, respecting the applicable regulations and prioritizing transmission of information based on scientific evidence.

## Data Availability

Os dados que sustentam este estudo estão disponíveis mediante solicitação ao autor correspondente, AD, pois, embora os dados tenham sido analisados de forma agregada e sem possibilidade de identificação individual (em consonância com a Resolução CNS 510/2016, Art. 1º, Parágrafo único, V), sua disponibilização controlada mediante solicitação visa preservar a privacidade e a confidencialidade dos pacientes.
