# Supplementary material for: Digital marketing in attracting new patients: cross-sectional study in a vascular surgery office
Source: J Vasc Bras. 2025 Dec 15;24:e20250042. doi: 10.1590/1677-5449.202500422 (PMC12704811; doi:10.1590/1677-5449.202500422)
Supplement: Translation

# Translation: *Marketing* digital na captação de novos pacientes: estudo transversal em um consultório de cirurgia vascular

**DOI:** 10.1590/1677-5449.202500421

**Authors:** Alex Lazzari Dornelles, Fabricio Rodrigues Santiago, Graciela Aparecida Pelegrini, Camila Biedler Giordani, Thais Muraro Simionato, Silvana Klaki Ternopilski, Mariana Mello

**Affiliations:** 1 Angioclínica, Chapecó, SC, Brasil.; 2 Clínica Longevittá – Angioclínica, Maravilha, SC, Brasil.; 3 Universidade Federal de Goiás – UFG, Goiânia, GO, Brasil.; 4 Instituto Federal de Santa Catarina – IFSC, Chapecó, SC, Brasil.; 5 Universidade de Passo Fundo – UPF, Passo Fundo, RS, Brasil.; 6 Universidade Federal do Rio Grande do Sul – UFRGS, Porto Alegre, RS, Brasil.

**Keywords:** mídias sociais, saúde, *marketing* de serviços de saúde, custos e análise de custo, tecnologia da informação em saúde

## Abstract

**Contexto:**

Com o passar dos anos e a evolução da tecnologia, as redes sociais se tornaram amplamente acessíveis, assumindo um importante papel nas relações interpessoais. Atualmente, é crescente a utilização dessas plataformas para a divulgação de informações referentes à saúde, que vão desde o diagnóstico até tratamentos e procedimentos médicos.

**Objetivos:**

Verificar o impacto da utilização das redes sociais na captação de novos pacientes em um consultório médico de angiologia e cirurgia vascular.

**Métodos:**

O estudo foi realizado em um consultório particular de cirurgia vascular, com uma amostra de 604 pacientes. Os dados foram coletados no período de janeiro de 2022 a dezembro de 2023, por meio de um cadastro eletrônico preenchido pela secretária no consultório.

**Resultados:**

De um total de 604 pacientes, 77% eram mulheres e 23% homens. Entre a amostra, 41% conheceram o consultório por meio de divulgações realizadas nas redes sociais. Isso gerou um impacto positivo de 38% no faturamento do consultório no período analisado.

**Conclusões:**

A análise dos resultados indicou que a presença do consultório nas redes sociais contribuiu positivamente para a captação de novos pacientes, bem como para o aumento do faturamento. O *marketing* digital é uma tendência que só tem a crescer com a difusão da internet, e incluir as redes sociais na divulgação de informações de saúde gera vantagens para ambas as partes: médico e paciente.

## INTRODUÇÃO

O *marketing* é uma importante ferramenta para diversas áreas, podendo ser aplicado a bens, serviços, eventos, experiências, pessoas, lugares, propriedades, organizações, informações e ideias^1^. Com a disseminação dos computadores e das tecnologias de informação, o *marketing* começou a desviar sua atenção para a internet, dando origem ao *marketing* digital (MD)^2^.

O MD refere-se à comercialização de produtos e serviços em formato digital^3^ e, na área da saúde, tornou-se um diferencial na prestação de serviços, permitindo que diferentes profissionais, inclusive os médicos, promovam seus serviços para pessoas em todo o mundo, e não apenas nas proximidades de seus consultórios^4,5^. Isso, por sua vez, possibilita que a divulgação de seu trabalho acompanhe as atuais tendências do cenário digital.

Com a internet, as redes sociais, os aplicativos móveis e outras tecnologias de comunicação digital integrados à vida cotidiana, bilhões de pessoas em todo o mundo utilizam essas ferramentas diariamente^6^, o que torna relevante a presença on-line de um consultório médico para fins de visibilidade e captação de novos pacientes. Acerca disso, é notável o aumento do uso de ferramentas de comunicação digital e das redes sociais por parte de diferentes profissionais da saúde, especialmente por médicos^7^.

Desde o surgimento das redes sociais, uma porcentagem crescente de pacientes utiliza essa tecnologia para questões relacionadas à saúde^8,9^. Nesse contexto, a publicidade nas redes sociais representa um campo em expansão para diferentes áreas da medicina^10-12^.

Hoje, existem muitas plataformas de redes sociais com diferentes estilos de uso^13^. Entre elas, estão Instagram®, Facebook®, X® (antigo Twitter®), YouTube®, blogues e comunidades de saúde on-line, que são populares entre os pacientes, sendo utilizadas para a busca e o compartilhamento de informações^14-16^. Além disso, a utilização dessas plataformas pode facilitar o envolvimento do paciente, o alcance da comunidade em geral, a educação e o aprendizado entre pares^12^.

As redes sociais são consideradas fatores influentes na relação entre médico e paciente, e a utilização do MD otimiza o envolvimento do paciente, impactando em sua escolha e o levando a uma eventual compra ou contratação de um produto ou serviço^17,18^.

Com a interação entre médico e paciente proporcionada pelas redes sociais, torna-se mais fácil a troca de informações e a criação colaborativa de conteúdo. Ou seja, médico e paciente atuam juntos para que a informação chegue nas redes sociais com maior precisão^14,15,19^.

Com base no exposto, este artigo teve como objetivo geral verificar o impacto da utilização das redes sociais na captação de novos pacientes em um consultório médico de angiologia e cirurgia vascular. Para isso, os objetivos específicos foram investigar a forma como os pacientes tiveram conhecimento sobre o consultório e identificar a representatividade dos pacientes oriundos das redes sociais no faturamento do consultório. A relevância do estudo reside no fato de que a área investigada é altamente especializada, motivo pelo qual os pacientes, muitas vezes, precisam de informações detalhadas e confiáveis para escolherem um profissional ou um consultório.

## MÉTODO

Este é um estudo transversal retrospectivo com dados obtidos prospectivamente. A coleta de dados foi realizada por meio de uma pesquisa quantitativa transversal. Os dados foram obtidos a partir dos cadastros eletrônicos dos pacientes. Ao todo, foram coletados dados de 604 pacientes, entre janeiro de 2022 e dezembro de 2023. O critério de inclusão foi todos os novos pacientes cadastrados no sistema de cadastro eletrônico da clínica entre janeiro de 2022 a dezembro de 2023. Já o de exclusão foi pacientes cadastrados anteriormente a 2022 (n = 5.718) ou posterior a dezembro de 2023 (n = 992). A Figura 1 apresenta o fluxograma do estudo.

**Figura 1:** Fluxograma do desenho do estudo.

O tamanho amostral ideal foi calculado com base em um nível de confiança de 95%, margem de erro de 5% e proporção estimada de 41%, resultando em uma necessidade mínima de 372 pacientes. Como o estudo contou com 604 pacientes, o tamanho da amostra foi considerado adequado^20^.

O MD do consultório é feito por meio das plataformas Instagram®, Youtube® e *site* próprio. Em outubro de 2024, no Instagram®, onde é realizada mídia paga, o consultório possui 232 mil seguidores, enquanto no Youtube® são mais de 390 mil inscritos no canal do consultório.

A escolha pelo consultório em questão deu-se por ele cumprir os critérios preestabelecidos, quais sejam: estar presente nas redes sociais; publicar ativamente; atingir ao menos 200 mil pessoas por meio dessas redes; e ter, no mínimo, 5 anos de atividade na cidade. Para tabulação e análise descritiva dos dados, foram utilizados os *softwares* Microsoft Excel® e Statistical Package for the Social Sciences.

O presente estudo não passou pelo Comitê de Ética em Pesquisa (CEP), pois, conforme a Resolução nº 510, “pesquisa com bancos de dados, cujas informações são agregadas, sem possibilidade de identificação individual” não precisa ser registrada nem avaliada pelo sistema CEP do Conselho Nacional de Ética em Pesquisa (BRASIL, 2016, Art. 1º, Parágrafo único, V), parâmetro no qual o estudo se encaixa, visto que as informações do banco de dados foram acessadas agregadas e sem possibilidade de identificação do paciente. Este estudo seguiu as diretrizes Strengthening the Reporting of Observational Studies in Epidemiology (STROBE)^21^.

## RESULTADOS

O consultório analisado neste estudo iniciou as atividades no ano de 2014 e atende, em média, 18 pacientes por dia, nos últimos 3 anos. Os atendimentos, realizados de quarta a sexta-feira, compreendem consultas, retorno de consultas e realização de procedimentos. Durante o período do estudo, o consultório atendeu apenas pacientes privados e de um único convênio. Foram analisados os dados de todos os 604 novos pacientes registrados durante o período estudado. A Figura 2 apresenta a classificação etária destes pacientes.

**Figura 2:** Faixa etária dos participantes.

Quando analisados em conjunto, a idade média dos pacientes foi de aproximadamente 49 anos. Para as mulheres, a média foi de 48 anos, enquanto, entre os homens, a idade média ficou em 51 anos. As idades dos pacientes homens variaram de 15 a 85 anos, e das pacientes mulheres, entre 19 e 89 anos.

A proporção de homens e mulheres que participaram do estudo é apresentada na Figura 3. Ao todo, foram registradas 466 mulheres e 138 homens respondentes. Os pacientes também indicaram a forma como tiveram conhecimento do consultório, conforme Tabela 1.

**Figura 3:** Gênero dos participantes, em %.

**Tabela 1:** Origem do encaminhamento de novos pacientes.

**Origem**	**2022**	**2023**	**Total**
Redes sociais	129	118	247
Familiares/ amigos	116	112	228
Outros médicos	44	53	97
Indicação do convênio	13	7	20
Outras formas	10	2	12
**Total**	312	292	604

Dos 604 novos pacientes, 247 (41%) chegaram até o consultório por meio das redes sociais, 228 (38%) através da indicação de familiares e/ou amigos, 97 (16%) por indicação de outros médicos, e apenas 20 (3%) por indicação do convênio. Além disso, 12 pacientes conheceram o consultório de outras formas que não foram detalhadas.

Na Tabela 2, são detalhadas as informações acerca dos pacientes provenientes das redes sociais. Percebe-se que as maiores concentrações de pacientes captados pelas redes sociais estão nas faixas de idade de até 58 anos. Os pacientes com idades entre 59 e 80 anos são os menos atingidos diretamente pelas informações postadas nas redes sociais do consultório.

**Tabela 2:** Detalhamento dos pacientes captados em relação às redes sociais.

**Faixa etária**	**Total na amostra**	**Redes sociais**	**% recrutados via mídias sociais em cada faixa etária**
Entre 15 e 25 anos	18	9	50,00
Entre 26 e 36 anos	129	61	47,29
Entre 37 e 47 anos	188	80	42,55
Entre 48 e 58 anos	100	43	43,00
Entre 59 e 69 anos	91	29	31,87
Entre 70 e 80 anos	52	16	30,77
Entre 81 e 91 anos	25	9	36,00
Não informou	1	0	0,00
Total	604	247	

Entre a amostra total, 156 pacientes possuem 60 anos ou mais. Destes, oito (1,32%) tiveram conhecimento do consultório por intermédio do convênio médico, 55 (9,10%) por indicação de familiares ou amigos, 50 (8,27%) pelas redes sociais, 38 (6,29%) pela indicação de outros médicos e cinco (0,82%) não souberam informar.

Também, durante o período de janeiro de 2022 a dezembro de 2023, foi realizado o controle para identificar o percentual de representatividade dos pacientes oriundos das redes sociais no faturamento mensal do consultório. Na Figura 4, é apresentada a informação referente ao ano de 2022.

**Figura 4:** Faturamento referente a pacientes vindos por meio das redes sociais no ano de 2022, em %.

Em 2022, o consultório teve 312 novos pacientes, sendo que, destes, 129 (41,34%) eram provenientes das redes sociais. No mesmo ano, o faturamento médio referente a pacientes vindos das redes sociais foi de 35%.

Na Figura 5, são apresentados os dados referentes ao ano de 2023, quando o consultório recebeu 292 novos pacientes, sendo 118 provenientes das redes sociais, o que representou, em média, 41% do faturamento.

**Figura 5:** Faturamento referente a pacientes vindos por meio das redes sociais no ano de 2023, em %.

## DISCUSSÃO

No Brasil, a área da saúde registrou um crescimento significativo na última década, com o número de médicos ingressando no mercado superando, de forma significativa, o número de profissionais que deixaram a atividade^22,23^. Entre os anos de 2010 e 2024, a população de médicos no Brasil passou de 304.406 para 575.930 indivíduos, e a previsão é de que, em 2035, a marca de 1 milhão de médicos seja ultrapassada, conforme dados da Demografia Médica no Brasil^23^. Na Figura 6, são apresentadas as entradas e saídas anuais de médicos, no período de 2010 até 2023.

**Figura 6:** Evolução das entradas e saídas de médicos registradas.

Esse cenário de crescente competição profissional na área^24^, aliado a um maior uso da internet e das redes sociais por parte da população brasileira^25,26^, cria um ambiente propício para a utilização do MD na área da saúde.

Uma recente pesquisa nacional^25^ identificou que 93,4% das pessoas utilizam a internet todos os dias para conversar por chamadas de voz ou vídeo (94,4%), enviar ou receber mensagens (92,0%), assistir vídeos (88,3%) e usar as redes sociais (83,6%), entre outros. Em janeiro de 2024, os 187,9 milhões de usuários de internet no Brasil estavam espalhados entre diversas plataformas. A Tabela 3 apresenta a quantidade de usuários nas três principais redes sociais, bem como o alcance dos anúncios nestas plataformas.

**Tabela 3:** Número de usuários e alcance dos anúncios nas principais redes sociais utilizadas no Brasil em 2024.

**Rede Social**	**Usuários**	**Alcance**	**Mulheres**	**Homens**
	**(milhões)**	**(%)**	**(%)**	**(%)**
YouTube	144,0	66,3	51,4	48,6
Instagram	134,6	62,0	58,4	41,6
Facebook	111.3	51,3	53,8	46,2

Fonte: Data Report Digital^26^.

Conforme relatório divulgado, Youtube®, Instagram® e Facebook® são as redes sociais mais utilizadas no Brasil. As mulheres representam mais da metade dos usuários dessas redes, que possuem significativo alcance nos anúncios. O alcance dos anúncios é calculado e apresentado em relação ao percentual total da população do Brasil^25^.

Para muitas pessoas, o uso de redes sociais se tornou uma parte significativa da vida diária e a principal fonte de informação^24-26^. Isso ressalta a importância da utilização da internet na comunicação de *marketing*, especialmente para alcançar os consumidores que a utilizam como meio de obter informação, se comunicar, se expressar e construir relacionamentos^27,28^.

As pessoas estão, cada vez mais, recorrendo à internet para buscar informações sobre diagnósticos, marcar consultas e conhecer mais sobre os médicos e suas especialidades^24,29^, sendo as redes sociais as plataformas preferidas para acesso imediato a informações^30^. Esses fatores destacam a importância da presença dos profissionais de saúde, especialmente os médicos, nas redes sociais. Além disso, o uso crescente das redes no cotidiano das pessoas^31^ e sua influência no comportamento do consumidor através do MD^3^ têm levado a um eventual consumo de produtos e serviços^18^.

Nos últimos anos, os profissionais da saúde passaram a utilizar cada vez mais as redes sociais para se comunicar com seus pacientes e promover a saúde^7,32^. A utilização do MD nas redes sociais permite aos profissionais demonstrar sua competência a pacientes em potencial, expandir sua área de atuação, melhorar a imagem do setor de saúde como um todo, alcançar um público maior e oferecer cuidados personalizados^5,33^.

Embora os médicos estejam utilizando cada vez mais as redes sociais, há ainda aqueles que hesitam em adotar esta prática^32^. Pesquisas recentes^9,12-14,30,32,34^ realizadas em diferentes áreas médicas investigaram o uso das redes sociais por médicos e ressaltaram aspectos positivos da prática. Entre os aspectos encontrados, estão o fato de facilitar a comunicação do paciente com o médico, a capacidade de aumentar a publicidade do médico e a promoção acelerada da saúde.

Analisando os dados referentes à amostra estudada, constatou-se que as formas mais frequentes pelas quais os pacientes tomaram conhecimento do consultório foram por meio das redes sociais e de familiares ou amigos (boca a boca). Acerca disso, pode-se afirmar que as redes sociais oferecem aos pacientes uma plataforma a partir da qual podem iniciar sua busca por um profissional^30^.

A presença nas redes sociais permite que os médicos se dirijam diretamente a um enorme coletivo de pacientes^10^, mas, como nem todas as plataformas são iguais e seu uso varia de geração para geração, é importante conhecer o perfil dos pacientes. O *marketing* eficaz em redes sociais deve considerar a entrega do conteúdo e a escolha da plataforma certa, variáveis que dependem da faixa etária específica do público que se deseja atingir^12^.

Entre as 466 mulheres da amostra deste estudo, 195 (41,85%) foram captadas por meio das redes sociais. Já entre os 138 homens, 52 (37,68%) tiveram conhecimento sobre o consultório por meio das redes sociais. O fato de o público feminino optar mais frequentemente pelo consultório devido às redes sociais pode estar associado a um levantamento realizado recentemente^26^, onde as mulheres representam mais da metade dos usuários, superando os homens. Nesse sentido, as mulheres podem ser mais receptivas ou mesmo buscar ativamente a interação com seu médico nas mídias sociais^34^. No caso de especialidades voltadas principalmente ao público feminino, esse é um dado bastante relevante.

Com relação à faixa etária, identificou-se que os pacientes entre 59 e 80 anos são os menos atingidos diretamente pelas informações postadas nas redes sociais do consultório. Esses pacientes com idade mais avançada podem ser influenciados pelos jovens, que tiveram acesso às informações ou que conheceram o consultório médico pelas redes sociais, visto que as novas gerações cresceram em um ambiente onde a mídia digital se tornou amplamente presente^35^.

Considerando dados publicados pelo Instituto Brasileiro de Geografia e Estatística (IBGE)^25^ referentes à utilização da internet no Brasil durante o ano de 2022, o percentual de idosos (60 anos ou mais) que utilizam a internet foi de 62,1%. Isso evidencia uma oportunidade de público que ainda pode ser atingido pelo consultório por meio do MD.

A partir dos dados que foram coletados, é possível personalizar os conteúdos para a faixa etária que se deseja contemplar. Nesse sentido, outros autores^12^ identificaram que as gerações mais jovens são mais propensas a utilizar o Instagram®, Snapchat® e TikTok®, enquanto as gerações mais velhas podem ser mais propensas a utilizar o Facebook® e o YouTube®^12^ para buscar informações sobre saúde.

A análise dos dados também indicou um impacto significativo no faturamento do consultório devido ao MD realizado, com presença no Instagram®, Youtube® e *site* próprio. Atendendo a pacientes privados e de apenas um convênio no período analisado, observou-se que, em média, as redes sociais representaram 35% do faturamento no ano de 2022 e 41% no ano de 2023. Apesar de o estudo compreender um curto período de tempo, percebe-se que há uma progressão constante nos números.

A representatividade das redes sociais pode ser ainda maior se considerarmos que, com as informações fornecidas pelo médico no Instagram®, Youtube® e no *site* próprio, os pacientes chegam ao consultório com mais conhecimento e predisposição à compra de produtos ou serviços. Isso pode ser explicado pela confiança que adquirem no médico devido a todas as informações que foram passadas por ele nas redes sociais.

Corroborando esse fato, outros autores^17^ afirmam que, quando em contato com conteúdos relacionados à saúde, as pessoas expressam preocupações sobre a legitimidade das informações e suas fontes e, portanto, tendem a confiar mais em informações fornecidas por médicos ou profissionais de saúde, em vez de em informações enganosas aleatórias.

Os números encontrados confirmam o benefício econômico de se utilizar as redes sociais, que também aumentam a publicidade do médico^10,12-14,30^. Além do impacto gerado pelos novos pacientes captados pelas redes sociais, é preciso considerar a economia de custos ao se comparar as campanhas publicitárias convencionais com aquelas realizadas por meio das redes sociais^10^.

Já em relação à amostragem do estudo, apesar da amostra utilizada ter sido robusta, foi realizado o cálculo do tamanho amostral ideal com base em um nível de confiança de 95%, margem de erro de 5% e proporção estimada de 41%^20^. O resultado indicou a necessidade mínima de 372 pacientes para garantir a representatividade dos dados. Como o estudo contou com 604 participantes, o número de pacientes superou o mínimo necessário, conferindo maior confiabilidade aos achados e fortalecendo a validade das conclusões. Assim, os resultados apresentados refletem com segurança o impacto positivo das redes sociais na captação de novos pacientes em consultórios de angiologia e cirurgia vascular.

Por fim, embora este estudo tenha demonstrado o impacto positivo do MD na captação de pacientes e no faturamento do consultório, é essencial considerar os aspectos éticos da propaganda médica^36,37^. De acordo com o Código de Ética Médica, a publicidade deve ser conduzida de forma responsável, evitando o sensacionalismo, a autopromoção ou a criação de expectativas irreais^37^. A divulgação nas redes sociais deve priorizar informações baseadas em evidências científicas, respeitando a privacidade dos pacientes e as regulamentações vigentes^36,37^. Quando realizado de maneira ética e transparente, o MD pode não apenas aproximar médicos e pacientes, mas também promover a educação em saúde e fortalecer a confiança na relação entre profissional e cliente.

## CONCLUSÃO

Com o aumento da concorrência no setor médico, o uso das redes sociais para MD tem se mostrado fundamental para atrair novos pacientes e destacar consultórios. Este estudo evidenciou que 41% dos novos pacientes do consultório analisado foram captados por meio dessas plataformas, demonstrando o impacto significativo do MD. Além disso, as redes sociais contribuíram para um aumento médio de 38% no faturamento do consultório.

Embora os resultados sejam promissores, é importante considerar as limitações do estudo, que foi realizado em um consultório particular de cirurgia vascular, onde os hábitos dos pacientes em relação ao uso das redes sociais podem não ser generalizáveis. No entanto, o MD em saúde tem um futuro promissor, com oportunidades de inovação e crescimento que podem beneficiar financeiramente os médicos e aumentar o reconhecimento de suas marcas. Por fim, é imprescindível que o MD em saúde seja conduzido de forma ética e responsável, respeitando as regulamentações vigentes e priorizando a transmissão de informações baseadas em evidências científicas.

## Data Availability

The data supporting this study are available upon request from the corresponding author, AD. Although the data were analyzed in an aggregated form with no possibility of individual identification (in accordance with Resolution CNS 510/2016, Article 1, Sole Paragraph, V), controlled access via request aims to protect patient privacy and confidentiality.
